# Mid-term clinical outcomes and cardiac function in patients receiving cardiac contractility modulation

**DOI:** 10.1007/s10840-024-01900-0

**Published:** 2024-08-29

**Authors:** Andrew Deak, Syed M. Zaidi, Chethan Gangireddy, Edmond Cronin, Eman Hamad, Carly Fabrizio, Sanjana Bhatia-Patel, Val Rakita, Isaac R. Whitman

**Affiliations:** Department of Medicine, Section of Cardiology, Parkinson Pavilion, 9th Floor, 3401 North Broad Street, Philadelphia, PA 19140 USA

**Keywords:** Heart failure, Devices, CCM, Cohort study

## Abstract

**Objectives:**

To describe the mid-term clinical and functional cardiac contractility modulation therapy (CCM) recipients in an urban population with heart failure.

**Background:**

CCM is a non-excitatory electrical therapy for patients with systolic heart failure with NYHA class III symptoms and ejection fraction (EF) 25–45%. How CCM affects a broad range of clinical measures, including diastolic dysfunction (DD) and weight change, is unexplored.

**Methods:**

We reviewed 31 consecutive patients at our center who underwent CCM implant. NYHA class, hospitalizations, ejection fraction (EF), diastolic function, and weight were compared pre- and post-CCM implant.

**Results:**

Mean age and follow-up time was 63 ± 10 years and 1.4 ± 0.8 years, respectively. Mean NYHA class improved by 0.97 functional classes (*p* < 0.001), and improvement occurred in 68% of patients. Mean annualized hospitalizations improved (0.8 ± 0.8 vs. 0.4 ± 1.0 hospitalizations/year, *p* = 0.048), and after exclusion of a single outlier, change in annualized days hospitalized also improved (total cohort 3.8 ± 4.7 vs. 3.7 ± 14.8 days/year; *p* = 0.96; after exclusion, 3.8 ± 4.7 vs. 1.1 ± 1.9 days/year, *p* < 0.001). Mean EF improved by 8% (*p* = 0.002), and among those with DD pre-CCM, mean DD improvement was 0.8 “grades” (*p* < 0.001). Mean weight change was 8.5 pounds lost, amounting to 4% of body weight (*p* = 0.002, *p* = 0.002, respectively), with 77% of patients having lost weight after CCM. Five patients (16%) experienced procedural complications; incidence skewed toward early implants.

**Conclusion:**

In an observational cohort, CCM therapy resulted in improvement in NYHA class, hospitalizations, systolic and diastolic function, and weight.

**Graphical Abstract:**

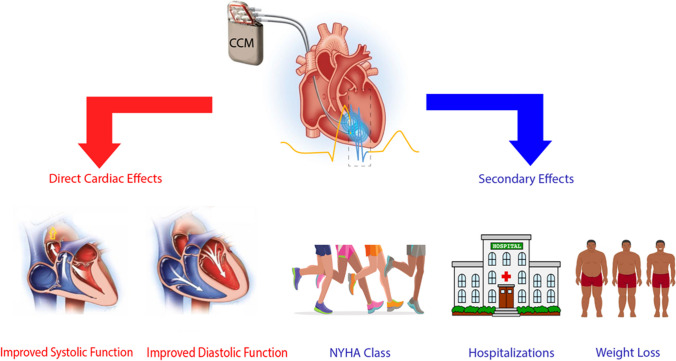

## Introduction

Cardiac contractility modulation therapy (CCM) is a novel electrical therapy for systolic heart failure (HF) patients with New York Heart Association (NYHA) class III symptoms, ejection fraction (EF) 25–45%, and who are not candidates for, or do not respond to, cardiac resynchronization therapy (CRT). The CCM device initiates non-excitatory electrical signals during the local ventricular absolute refractory period to modify the biology of the failing cardiac muscle on the molecular, cellular, and extracellular levels, including improving myocyte contractile force without increasing myocardial oxygen consumption [5, 10, 19]. CCM may improve symptoms, reduce hospitalizations, and increase EF in a controlled clinical trial setting [4]. However, outside of clinical trials, there is a dearth of real-world data as to how CCM affects patient outcomes. In this study, we address this knowledge gap by describing our single-center urban experience with CCM, specifically observing effects on functional status, hospitalization, EF, diastolic dysfunction (DD), and weight loss.

## Methods

### Study population

We retrospectively reviewed the charts of 31 consecutive patients who had undergone CCM placement at our urban, quaternary care institution between 2020 and 2023. The study was approved by the Temple University Hospital Institutional review board.

### Data collection and measurement

Clinical information including functional status, patient symptoms, hospitalization data, weight, and echocardiogram reports were collected from the electronic medical record (Epic Systems®, Madison, Wisconsin). All patients followed with outpatient cardiologists (both heart failure and electrophysiology specialists), and NYHA classes were determined by functional status documented at those appointments. Hospitalizations were inpatient admissions characterized as HF hospitalizations or potential heart HF hospitalizations (e.g., “shortness of breath”), arrhythmia-related, procedural complications, or cardiac device sequela. All hospitalizations due to other medical pathologies or symptoms unrelated to HF or cardiac devices were not included. Hospitalizations were included if they occurred after the first measured EF < 45%, or first charted diagnosis of “HF,” whichever came first. Days hospitalized were determined by “hospital days” listed on each patient’s discharge summary. All EFs were determined by transthoracic echocardiogram and recorded as the high-end estimate in any EF range. All echocardiograms were done > 3 months after implant, and the last echo during the follow-up period was used for the analyses. Weight was collected at clinic visits, regardless of medical specialty, and the most recent weight at the end of the follow up period was used to determine weight change. Weight change data was determined by examining each patient’s total follow up time after CCM placement, then using the closest documented weight to that same individualized duration of time prior to CCM placement for initial weight prior to CCM. Normally distributed continuous variables were reported as means ± standard deviation and were compared using Student’s *t* test.

## Results

Of the 31 consecutive patients reviewed, demographics and clinical characteristics are shown in Table 1. Mean age was 63 ± 10 years, most patients were Black, nearly half were women, half had non-ischemic cardiomyopathy (NICM) (*n* = 15, one with hypertrophic cardiomyopathy, the rest with idiopathic cardiomyopathy), and 68% had carried the diagnosis of cardiomyopathy (CM) for > 3 years. Mean follow-up time was 1.4 ± 0.8 years after CCM implant.

**Table 1:** Summary statistics and individual patient characteristics

Patient number	Age by decade (years)	Sex (M/F)	Race	Type of CM	Duration of CM prior to CCM implant > 3 years (Y/N)	HTN (Y/N)	DM (Y/N)	EF at time of CCM implant (%)	ICD at time of CCM implant (Y/N)	BMI (kg/m^2^)
*Summary statistics*	63 ± 10 years	23 (74) Male	15 (48) Black, 7 (23) Hispanic, 6 (19) White, 3 (10) Other*	16 (52) ICM, 14 (45) NICM, 1 (3) HCM	21 (68) > 3 Years	28 (90) with HTN	19 (61) with DM	32 ± 7	22 (71) with ICD	34.8 ± 5.7
1	40–49	M	Hispanic	ICM	N	N	Y	25	Y	39
2	40–49	M	Hispanic	ICM	N	Y	Y	30	Y	30.3
3	50–59	F	Hispanic	NICM	N	N	N	25	Y	33.3
4	50–59	M	Black	NICM	Y	Y	Y	30	Y	38.8
5	50–59	M	Black	NICM	N	Y	N	35	Y	29.3
6	50–59	M	Black	NICM	Y	Y	Y	35	Y	51.7
7	50–59	M	Hispanic	ICM	Y	Y	Y	40	Y	32.3
8	50–59	M	White	ICM	Y	Y	N	35	Y	33.6
9	50–59	M	Hispanic	ICM	Y	Y	N	30	Y	45.3
10	50–59	M	Hispanic	ICM	N	Y	Y	25	Y	40.5
11	50–59	M	Other	NICM	N	Y	Y	35	N	31.3
12	60–69	F	Black	NICM	Y	Y	Y	40	N	30.4
13	60–69	F	Black	NICM	N	Y	Y	40	Y	35.7
14	60–69	M	Black	ICM	Y	Y	Y	35	Y	31.9
15	60–69	M	Black	NICM	Y	Y	Y	25	Y	40.8
16	60–69	M	White	ICM	Y	Y	Y	25	Y	31.3
17	60–69	M	Black	NICM	Y	Y	Y	40	N	34.9
18	60–69	F	Hispanic	NICM	N	Y	N	40	N	29.6
19	60–69	F	Black	ICM	Y	Y	Y	50	N	39.1
20	60–69	F	White	HCM	Y	Y	N	35	Y	31.8
21	60–69	M	Other	NICM	Y	N	N	25	Y	42.6
22	70–79	M	Black	ICM	Y	Y	Y	30	Y	34
23	70–79	M	Other	ICM	Y	Y	N	25	Y	32.2
24	70–79	M	Black	NICM	N	Y	N	25	Y	33.5
25	70–79	F	Black	ICM	Y	Y	N	35	N	26.6
26	70–79	F	White	NICM	Y	Y	Y	40	N**	37.6
27	70–79	M	White	ICM	N	N	N	35	N	31
28	70–79	M	White	ICM	Y	Y	N	25	Y	36.9
29	70–79	M	Black	NICM	Y	Y	Y	40	N	38.6
30	80–89	M	Black	ICM	Y	Y	Y	45	Y	24.5
31	80–89	M	Black	ICM	Y	Y	Y	20	Y	29.3

*Other: self-reported in the institutional electronic medical record whereby pre-specified race categories did not apply

**Met indications for primary prevention defibrillator but declined implant. Summary statistics: data provided as mean ± standard deviation or *n* (%)

*BMI*, Body mass index; *CM*, cardiomyopathy; *CCM*, cardiac contractility modulation; *CKD*, chronic kidney disease; *DM*, diabetes; *EF*, ejection fraction; *F*, female; *HTN*, hypertension; *ICD*, implantable cardioverter-defibrillator; *ICM*, ischemic cardiomyopathy; *M*, male; *NICM*, non-ischemic cardiomyopathy

Age provided as ranges and chronology of patients listed are random to protect patient privacy given the small number of patients and single center observation

### Exclusions

Several patients were excluded from specific analyses (Table 2). One patient, after receiving CCM, died 609 days later of progressive HF, which included cardiac cachexia and inpatient death (excluded from ex post facto hospitalizations and weight change analyses). One patient, who had an ischemic cardiomyopathy, prior ischemic stroke, and atrial fibrillation on anticoagulation, died in his sleep 39 days after CCM (excluded from echocardiogram-related endpoints). Two other patients were excluded from echocardiogram-related endpoints due to failure to obtain repeat echocardiograms by the time of the present analyses.

**Table 2:** Characteristics of patients excluded from specific analyses

Age by decade (years)	Type of CM	BMI at CCM implant (kg/m^2^)	Reason for exclusion	Analysis exclusions
50–59	NICM	37.1	Extreme outlier: Progressive HF leading to cardiac cachexia, 10 inpatient hospitalizations, 177 days hospitalized over a 2-year period, and eventual inpatient death	Hospitalization and weight analyses
50–59	NICM	31.3	Follow-up echocardiogram not completed	EF and DD analyses
70–79	ICM	36.2	Sudden cardiac death less than 40 days after CCM placement; follow-up echocardiogram not completed	EF and DD analyses
70–79	NICM	36.5	Follow-up echocardiogram not completed	EF and DD analyses

*BMI*, Body mass index; *CM*, cardiomyopathy; *CCM*, cardiac contractility modulation; *DD*, diastolic dysfunction; *EF*, ejection fraction; *HF*, heart failure; *ICM*, ischemic cardiomyopathy; *NICM*, non-ischemic cardiomyopathy

Age provided as ranges to protect patient privacy given the small number of patients and single center observation

### New York Heart Association class

**Fig. 1:**
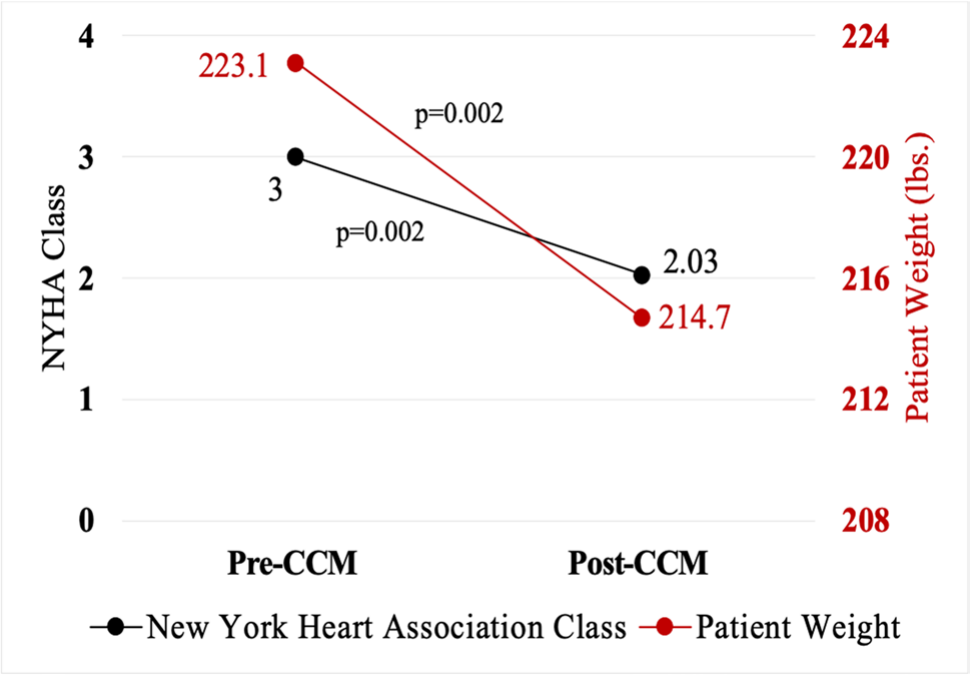
Change in functional class and weight after cardiac contractility modulation. Mean change in weight was − 8.5 ± 14.9 pounds (*p* = 0.002), and mean change in NYHA class was an improvement of 0.97 functional classes (*p* = 0.002). Weight loss, in the setting of improving cardiac function, may be a product of increased activity and/or improvements in volume status after CCM placement. Improved NYHA functional class is associated with decreased risk of all-cause mortality and improvements in overall quality of life. CCM, cardiac contractility modulation; NYHA, New York Heart Association

NYHA class improvement occurred in 68% (*n* = 21) of patients. All patients were NYHA class III at the start of the study, the mean improvement was 0.97 functional classes (*p* < 0.001), and 29% (*n* = 9) experienced improvements in NYHA class of greater than one functional class (Fig. 1). One patient (3%) had a worsening in NYHA functional class. Of the 21 patients with NYHA class improvements, 52% (*n* = 11) had NICM, and 48% (*n* = 10) had ischemic cardiomyopathy (ICM).

### Annual hospitalizations and days hospitalized

**Fig. 2:**
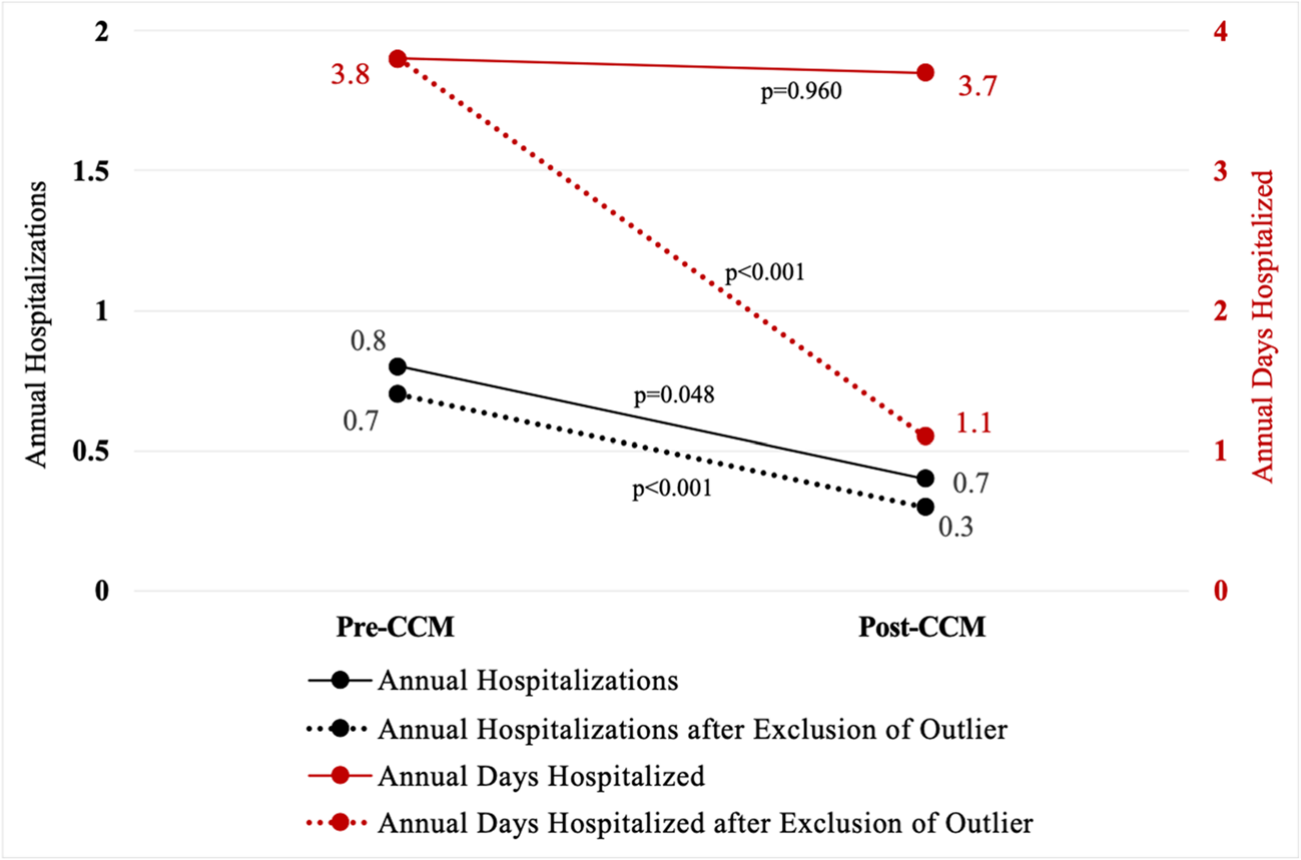
Change in hospitalization data after cardiac contractility modulation. After exclusion of a single outlier, annual hospitalizations (0.7 ± 0.7 hospitalizations/year vs. 0.3 ± 0.6 hospitalizations/year, *p* < 0.001), and annualized hospital days per year were lower after CCM (3.8 ± 4.7 days/year vs 1.1 ± 1.9 days/year, *p* < 0.001). A reduction in hospitalizations and days hospitalized represents a reduction in morbidity. CCM, cardiac contractility modulation

When comparing the time from HF diagnosis through CCM placement to the time from CCM implant through follow-up, there was a reduction in mean annualized hospitalizations (0.8 ± 0.8 hospitalizations/year pre-CCM vs. 0.4 ± 1.0 hospitalizations/year post-CCM, *p* = 0.048). Annualized days hospitalized was not statistically different pre- and post-CCM (3.8 ± 4.7 days/year pre-CCM; 3.7 ± 14.8 days/year post-CCM; *p* = 0.960). In an ex post facto sub-analysis excluding the single outlier, there was a more marked reduction in annual hospitalizations (0.7 ± 0.7 hospitalizations/year vs. 0.3 ± 0.6 hospitalizations/year, *p* < 0.001), and annualized hospital days per year was also lower after CCM (3.8 ± 4.7 days/year vs 1.1 ± 1.9 days/year, *p* < 0.001) (Fig. 2). Patients with no change in annual hospitalizations and days hospitalized, of which there were six (19% of total cohort), were not hospitalized prior to or after CCM placement. Two patients (7%) had an increase in hospitalizations and/or days hospitalized after CCM when compared to prior to CCM (including the single outlier). Although not statistically significant, point estimates for improvement in annual hospitalizations and annual days hospitalized was greater in patients who had CCM implant within 3 years of HF diagnosis when compared to those with CCM placed more than 3 years after HF diagnosis (change in hospitalizations: − 0.6 ± 0.7 hospitalizations/year in those with CCM within < 3 years vs − 0.2 ± 0.9 hospitalizations/year in those with CCM after > 3 years, *p* = 0.27; change in annual hospital days: − 2.9 ± 3.3 days/year in those with CCM within < 3 years vs + 1.2 ± 18.2 days/year in those with CCM after > 3 years, *p* = 0.48). Of the 21 patients with NYHA improvements outlined above, none had an increase in hospitalizations after CCM.

### Systolic and diastolic function

**Fig. 3:**
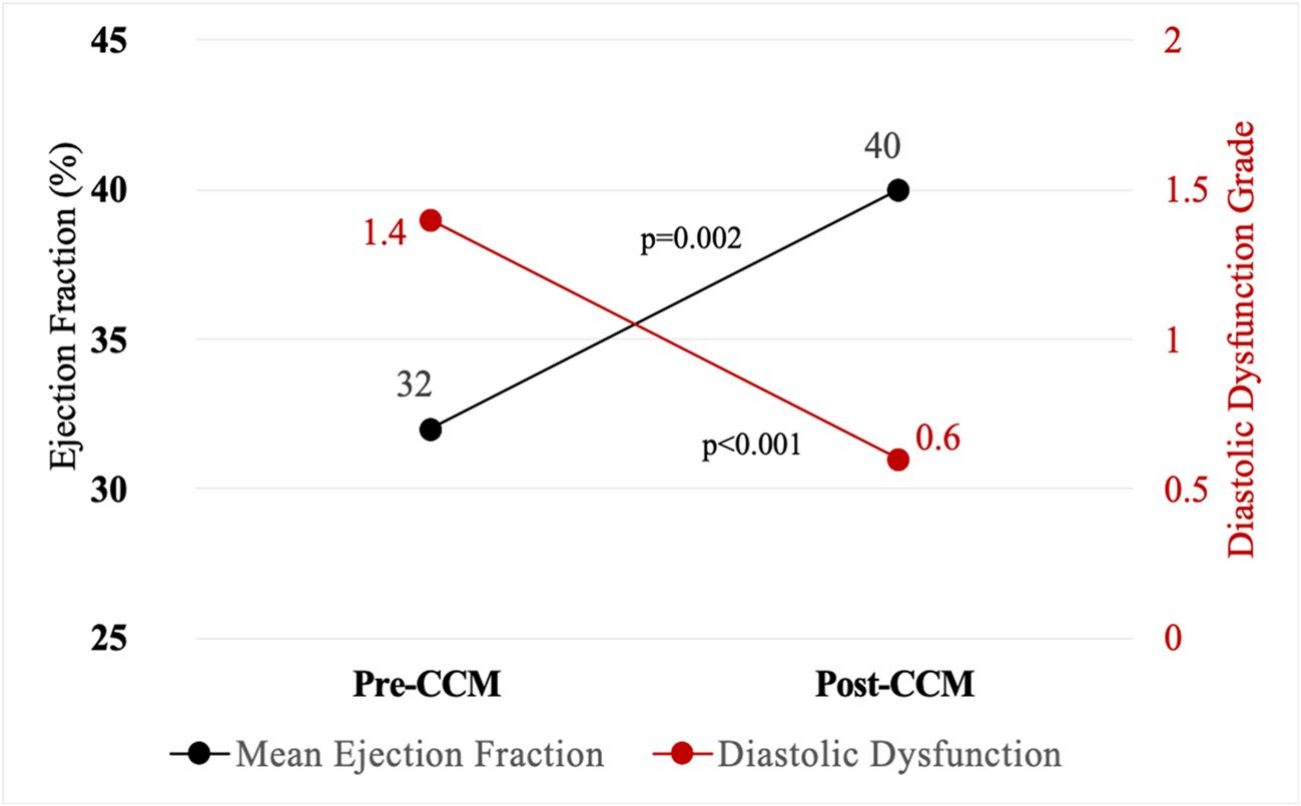
Changes in systolic and diastolic function after cardiac contractility modulation. Mean EF improved by 8% (*p* = 0.002), and using a scale of DD from grade 1 to 3, mean DD improved by 0.8 grades (*p* < 0.001). Improvements in EF are consistent with prior literature on CCM. DD improvement suggests CCM affects the heart in multiple ways, including positive effects on ventricular myocardial stiffness. CCM, cardiac contractility modulation; DD, diastolic dysfunction; EF, ejection fraction; ICD, implantable cardioverter-defibrillator

Mean EF improved by 8% (pre-CCM 32% ± 7% vs. post-CCM 40% ± 12%, *p* = 0.002). A total of 68% (*n* = 19) experienced EF improvement, with 25% (*n* = 7) demonstrating an EF improvement of 15% or greater (Fig. 3). Of the 19 patients who experienced an EF improvement, 53% (*n* = 10) had ICM, and 47% (*n* = 9) had NICM. No change in EF was observed in 18% (*n* = 5), and 14% (*n* = 4) had a decrement in EF. Interestingly, of the 20 patients who met criteria for primary prevention ICD at the time of CCM implant, 40% (*n* = 8) experienced an EF improvement to > 35%.

At baseline, DD was present in 79% of patients (*n* = 22), with 41% (*n* = 9) having NICM and 59% (*n* = 13) ICM. With DD graded on a scale from 1 to 3, the mean improvement was 0.8 grades (*p* < 0.001) (Fig. 3). Of patients with baseline DD, 55% (*n* = 12) had improvement in diastolic function after CCM; and of these 12, 42% (*n* = 5) had NICM, and the rest (58%, *n* = 7) had ICM. One patient developed worsened DD in follow-up. The improvements in DD did not correlate with improvements in systolic function (EF) in this study (correlation coefficient =  − 0.196, *p* = 0.38). Of the 21 patients with NYHA class improvement, 18 patients had follow-up echocardiograms completed at the time of this analysis. Improvement in EF was seen in 78% (*n* = 14), improvement in DD was seen in 44% of the entire cohort (*n* = 8), and 83% (*n* = 15) had improvement in EF and/or DD.

### Weight change

In the individualized time periods before CCM placement, which was equal to the total follow up time after CCM placement for each patient, mean weight change was + 5.1 ± 24.4 pounds (2.1% of total body weight) (*p* = 0.26 and *p* = 0.29 respectively), and 63% (*n* = 19) of patients gained weight. Over the same individualized time-period following CCM placement, 17% (*n* = 5) gained weight. However, mean weight change after CCM, when compared to weight at CCM placement, was − 8.5 ± 14.9 pounds, equivalent to 4.0% reduction in weight (*p* = 0.002 for both), with 77% (*n* = 23) of patients losing weight (Fig. 1). Comparing pre-CCM average weight change (+ 5.1 ± 24.4 pounds) to post-CCM weight change (− 8.5 ± 14.9), the data showed a mean weight change of − 13.5 ± 21.2 pounds. In the patients who lost weight following CCM, 57% (*n* = 13) were ICM, and 43% (*n* = 10) were NICM. In the group of 21 patients with improvements in NYHA functional status, 71% (*n* = 15) experienced new or accelerated weight loss after CCM placement.

### Complications

Several patients suffered complications after CCM implant (Table 3). Two patients (6%) died during follow-up (at 39 days post-CCM and 609 days post-CCM), and 5 patients (16%) had a procedural complication. The most common complication was pain related to CCM-therapy (*n* = 2). Most of the peri-procedural complications occurred earlier in the investigation period, suggesting a learning curve in the placement procedure. As the most common complication was pain, potential to decrease complication rate should be centered around lead placement to avoid the epicardial pain one can experiences with the high output of CCM therapy.

**Table 3:** Procedural complications after cardiac contractility modulation implant

Age by decade (years)	Type of CM	BMI at CCM implant (kg/m^2^)	Procedural complications
40–49	ICM	38.1	Superficial surgical site infection, resolved after oral antibiotics
50–59	NICM	51.7	Epicardial pain from CCM therapy requiring single lead revision
60–69	HCM	31.8	Ipsilateral shoulder pain necessitating device removal
70–79	NICM	33.2	Epicardial pain from CCM therapy and ventricular under-sensing requiring pocket and lead revision
70–79	ICM	36.9	Pneumothorax requiring chest tube, discharged next day on room air

*BMI*, Body mass index; *CM*, cardiomyopathy; *CCM*, cardiac contractility modulation; *HCM*, hypertrophic cardiomyopathy; *ICM*, ischemic cardiomyopathy; *NICM*, non-ischemic cardiomyopathy

Age provided as ranges to protect patient privacy given the small number of patients and single center observation

## Discussion

Device therapy is an important adjunct to medical heart failure therapy. In a group of 31 consecutive HF patients who underwent CCM implant at our hospital, we observed improvement in a meaningful proportion of patients on several fronts: NYHA class, hospitalizations and hospitalized days, systolic and diastolic function, and weight reduction. Based on our single-center experience, CCM may provide an opportunity to augment the care of certain heart failure patients.

### NYHA class

NYHA class improvement of at least one class occurred in over 65% of the patients in this study. This is relatively consistent with prior studies, although our proportion improved is slightly less than what was observed in clinical trials and large registries [3–5]. That the proportion with NYHA class improvement was slightly lower than previously published may represent simple variation across populations, may be related to differences in comorbidities, or a limitation of our small sample size. However, as it is well documented that impoverished communities have worse HF outcomes, it is worth noting that this study represents data from one of the nation’s most destitute communities, at what is the hospital providing the most charity care in the USA [22, 26, 30]. Nonetheless, improved NYHA functional class is associated with lower risk of hospitalization, lower risk of all-cause mortality, as well as improvements in overall quality of life, and has been used as a benchmark endpoint for clinical improvement in HF [15].

### Annual hospitalizations and days hospitalized

Heart failure is the most common reason for hospitalization among Medicare patients and the second most common overall, resulting in over 1 million hospitalizations per year in the USA [24]. Mortality rates for Medicare patients is between 15 and 20% within 90 days of hospitalization, and all patients with depressed EF, regardless of age, have a 5-year mortality of 69%, and median survival of 6 months [7, 17]. Therefore, reduction in hospitalizations is its own indication of reduction in morbidity and may be a surrogate measure of other untoward outcomes. CCM therapy has previously been shown to reduce hospitalizations, and our findings are consistent with previously demonstrated reductions in HF hospitalizations [1, 3–5, 18]. Although the point-estimates were not significantly different, we observed lower annual hospitalizations and annual days hospitalized in patients who had CCM placed more proximate to HF diagnosis, which aligns with CRT literature describing improved outcomes if device placement occurs prior to initial HF hospitalization, with incrementally worse outcomes as time passes from index hospitalization to ultimate device implant [18, 20]. This suggests that it is not just the HF intervention itself (device or otherwise) that is critical, but also its timing that may have important implications regarding outcomes. Preventing hospitalizations is a central tenet in the therapy of HF patients, as well as being a focus of improvement to reduce healthcare costs [14, 30].

### Systolic improvement

The EF improvement demonstrated in this observational study was predictable based on prior literature, including in both ICM and NICM patients [3, 9]. A potential implication of the EF improvement with CCM is whether CCM-therapy could render primary prevention ICD indication moot in some patients, who with CCM, experience improvement of EF to > 35%. Nonetheless, in CRT and non-CRT heart failure populations, recovery of EF does not normalize the risk of sudden death [32, 34]. There is no data that EF improvement in CCM patients reduces the risk of sudden death, and in fact, there was early concern that the increased intracellular myocardial calcium observed with CCM therapy could increase arrhythmogenicity. Ultimately, this hypothesis was not borne out in safety and then subsequent clinical studies, perhaps because CCM increases systolic, rather than diastolic calcium stores [1, 2, 6, 16, 28, 29]. It is important to emphasize, though, that just as patients with recovery of EF after primary prevention ICD remain at higher risk for ventricular arrhythmias than the general population, there is no data to suggest that those with EF recovery related to CCM would fair differently [23]. Certainly, the current study is too small to evaluate ICD therapies or ventricular arrhythmias as an outcome—although none of the patients experienced ICD therapy for ventricular arrhythmias, yet one patient without an ICD did die in his sleep 1 month after CCM implant. Our observation that 40% of ICD candidates (EF ≤ 35% after 3 months of GDMT) experienced an improvement in systolic function to EF > 35% is provocative, although the clinical application of these findings requires rigorous study before suggesting that ICDs be withheld in this subgroup.

### Diastolic improvement

CCM therapy has a lusitropic effect at the cellular level, and in an initial safety trial in patients with heart failure with preserved EF, was shown to be safe and to improve diastolic filling index (E/E’) [21]. Certainly, improvements in diastolic function are important, as improvements are associated with decreases in hospitalizations and mortality among those with HF [8]. In our study, more than half of those with DD had improvement in diastolic filling index, suggesting CCM therapy could have a positive effect on diastolic function in the clinical setting. Improved diastolic function did not correlate with improvements in EF nor with type of cardiomyopathy. Notably, in patients with improved NYHA class, 78% had improvement in EF, 44% had improvement in DD, and 28% had improvement in both. Among a small group of patients, it is inappropriate to draw firm conclusions, but it may be that CCM affects myocytes in multiple ways, which may manifest differently, depending on the heart. For example, as provocative conjecture, perhaps it is that diastolic improvements are at play in the increased benefit CCM has been shown to provide in those with LVEF 35–45% [2]. Bigger studies with similar granular data on the relationship between systolic and diastolic function may be clarifying.

### Weight loss

Although weight loss can be a poor prognostic indicator for worsening HF (cardiac cachexia), which was evident in the outlying patient, in this study, most of the studied patients showed weight loss to go along with other clinical improvements [31, 33]. A significant portion of patients had substantial weight loss after CCM, and unsurprisingly, the five patients who experienced improvement in NYHA class, hospitalizations, systolic, and diastolic function, all had meaningful weight loss. Weight loss, in the setting of improving functional capacity and overall cardiac function, may be a product of increased activity and/or improvements in volume status after CCM placement—and may portend better outcomes [25].

### Clinical worsening after CCM

Despite large proportions of patients demonstrating improvement in several endpoints, it is important to highlight that several patients did not improve. In the CRT population, so-called “non-responders” comprise approximately 30% of those treated, with some proportion of failures to respond related to insufficient GDMT, limited coronary sinus branch anatomy, and failures in device programming [11, 27]. But in CCM, where anatomy may not be as relevant and programming far more simplistic, it interesting to consider why some benefit, while others do not. One patient in our experience did profoundly poorly—spending more time in the hospital than out of it, and ultimately died of progressive heart failure, all after CCM implant. There were no signs prior to CCM to suggest this patient would not be a good candidate. The patient was adherent to all medications, had a BMI of 33.8 kg/m^2^, an EF of 35%, a left ventricular end diastolic dimension of 50 mm, and an estimated glomerular filtration rate of 50 mL/min/1.73 m^2^. He had suffered an out of hospital ventricular fibrillation arrest, aborted by therapy from his ICD, approximately 2 weeks prior to CCM implant. A consideration for his progressive disease may be the chronicity of his cardiomyopathy (first diagnosed 4 years prior to CCM implant); however, others had similar lead-times prior to CCM implant who fared better. Another patient with EF decline was our single dialysis-dependent patient. Dialysis patients may be a specific group of patients in whom the risk/benefit must be very carefully considered, given their increased risk of infection with implanted cardiac devices [12]. Alternative studies may have been performed as a matter of protocol, such as cardiopulmonary exercise testing or 6-min walk test, to help identify which patients are more likely to benefit from CCM.

#### Limitations

The limitations of this study relate to its small, uncontrolled, and unblinded nature. All measured outcomes were unblinded, and vulnerable to bias. Improvements in measured outcomes may have been related to CCM, or other unmeasured changes, like improvement in GDMT or improved vigilance of care in patients who had undergone what was a novel procedure at our institution. The lack of uniformity in follow-up time, as well broad period over which implants took place (2020–2023), may have resulted in differences in management, including that SGLT2 inhibitors and sacubitril/valsartan to replace ACEi/ARB in NYHA class III HF patients did not become part of societal guideline recommendations until 2022 [13]. Echocardiograms were performed at varying time intervals, and therefore, potential fluctuations of EF, in either direction, may not have been identified. Additionally, echocardiogram interpretations were performed by variable attending readers. Patients did not uniformly undergo quantitative testing of functional capacity before and after, such as a 6-min walk test and cardiopulmonary exercise testing. Weight data was collected from varying appointments, different scales, and at non-uniform intervals post-CCM.

## Conclusion

After implant of clinically indicated CCM devices, an observational cohort at a single, urban institution demonstrated, on average, improved NYHA class, decreased hospitalizations, improved systolic and diastolic function, and patient weight loss.

## Data Availability

All datasets are available via RedCap at the Temple University Hospital.

## Abbreviations

GDMT: Guideline-directed medical therapy
BMI: Body mass index
ACEi: Angiotensin-converting enzyme inhibitor
ARB: Angiotensin receptor blockers
SGLT2: Sodium-glucose cotransporter
